# Epigallocatechin-3-gallate-mediated cardioprotection by Akt/GSK-3β/caveolin signalling in H9c2 rat cardiomyoblasts

**DOI:** 10.1186/1423-0127-20-86

**Published:** 2013-11-19

**Authors:** Shih-Ron Hsieh, Chen-Sen Hsu, Chen-Hua Lu, Wei-Cheng Chen, Chun-Hwei Chiu, Ying-Ming Liou

**Affiliations:** 1 Department of Cardiovascular Surgery, Taichung Veterans General Hospital, Taichung 407, Taiwan; 2 Department of Life Sciences, National Chung-Hsing University, Taichung 402, Taiwan; 3 Institute of Bioinformatics and Structural Biology, National Tsing Hua University, Hsinchu 30013, Taiwan; 4 Graduate Institute of Basic Medical Science, China Medical University, Taichung 40402, Taiwan

**Keywords:** EGCg, Cell cycle, H9c2, EGFP, Caveolin, Oxidative stress

## Abstract

**Background:**

Epigallocatechin-3-gallate (EGCg) with its potent anti-oxidative capabilities is known for its beneficial effects ameliorating oxidative injury to cardiac cells. Although studies have provided convincing evidence to support the cardioprotective effects of EGCg, it remains unclear whether EGCg affect trans-membrane signalling in cardiac cells. Here, we have demonstrated the potential mechanism for cardioprotection of EGCg against H_2_O_2_-induced oxidative stress in H9c2 cardiomyoblasts.

**Results:**

Exposing H9c2 cells to H_2_O_2_ suppressed cell viability and altered the expression of adherens and gap junction proteins with increased levels of intracellular reactive oxygen species and cytosolic Ca^2+^. These detrimental effects were attenuated by pre-treating cells with EGCg for 30 min. EGCg also attenuated H_2_O_2_-mediated cell cycle arrest at the G1-S phase through the glycogen synthase kinase-3β (GSK-3β)/β-catenin/cyclin D1 signalling pathway. To determine how EGCg targets H9c2 cells, enhanced green fluorescence protein (EGFP) was ectopically expressed in these cells. EGFP-emission fluorescence spectroscopy revealed that EGCg induced dose-dependent fluorescence changes in EGFP expressing cells, suggesting that EGCg signalling events might trigger proximity changes of EGFP expressed in these cells.

Proteomics studies showed that EGFP formed complexes with the 67 kD laminin receptor, caveolin-1 and -3, β-actin, myosin 9, vimentin in EGFP expressing cells. Using in vitro oxidative stress and in vivo myocardial ischemia models, we also demonstrated the involvement of caveolin in EGCg-mediated cardioprotection. In addition, EGCg-mediated caveolin-1 activation was found to be modulated by Akt/GSK-3β signalling in H_2_O_2_-induced H9c2 cell injury.

**Conclusions:**

Our data suggest that caveolin serves as a membrane raft that may help mediate cardioprotective EGCg transmembrane signalling.

## Background

Green tea polyphenols (GTPs) have potent antioxidant and radical-scavenging properties, which may partially account for their cardioprotective effects [1]. The major catechins in GTPs include epicatechin (EC), epigallocatechin (EGC), epicatechin-3-gallate (ECG), and epigallocatechin-3-gallate (EGCg) [1,2]. EGCg is the most physiologically potent compound, and primarily accounts for the biological effects of green tea. Two recent reports using two different rat myocardial ischemic models of MI (myocardial infarction) [3] and IR (ischemia reperfusion) [4] associated with left anterior descending (LAD) coronary artery ligation have demonstrated that GTPs can efficiently improve cell viability during myocardial ischemic injury. Other studies of myocardial injury have also suggested that the cardioprotective effect of GTPs is associated with the scavenging of active-oxygen radicals, the modulation of redox-sensitive transcription factors (e.g., NFκB, AP-1), the reduction of STAT-1 activation and Fas receptor expression, an increase in NO production, and the exertion of positive inotropic effects [5-9]. Although studies have provided convincing evidence to support the cardioprotective effects of GTPs, it remains unclear whether GTPs affect trans-membrane signalling in cardiac cells.

A growing body of evidence has demonstrated that multiple signal transduction events for cardioprotection are mediated via signalling microdomains, such as lipid rafts or caveolae, on the plasma membrane of cardiac cells [10,11]. Caveolae are a subset of lipid rafts enriched in the protein caveolin (Cav) [12]. There are three isoforms of Cav, Cav-1, Cav-2 and Cav-3 [13], each of which functions as a scaffolding protein to organize and regulate membrane receptors and lipid-modified signalling molecules [14-16]. Cav-3 is the muscle-specific isoform in cardiac myocytes, whereas Cav-1 and Cav-2 are present in other cell types in the heart [17]. A study using in vitro and in vivo models of myocardial injury demonstrated that modification of the membrane structure and composition triggers Src activation and Cav-1 phosphorylation, resulting in cardioprotection [18]. More recently, another study with Cav-3 knock-out mice subjected to IR injury has shown that the expression of Cav-3 in cardiac myocytes is essential for isoflurane-induced cardioprotection from myocardial ischemic injury [19]. These data also suggested that Cav may mediate the beneficial actions of a variety of cardioprotective agents [19].

In this study, we examined the potential mechanism for EGCg-mediated cardioprotection in an H_2_O_2_-induced oxidative stress model of myocardial ischemia injury using H9c2 rat cardiomyoblasts. We first verified that the cardioprotection of EGCg is mediated by decreasing reactive oxygen species (ROS) and cytosolic Ca^2+^ and by preventing alterations in the protein expression of the adherens molecules β-catenin and N-cadherin and the gap junction protein connexin 43 (Cx43) in cardiac cells. In addition, EGCg was found to prevent H_2_O_2_-induced cell cycle arrest at G1-S phase via the glycogen synthase kinase-3β/β-catenin/cyclin D1 signalling pathway. To further clarify the putative mechanism underlying EGCg transmembrane signalling in cardiac cells, enhanced green fluorescence protein (EGFP) was ectopically expressed in H9c2 cells. EGFP-emission fluorescence spectroscopy indicated that Triton X-100-resistant microdomains (i.e., lipid rafts) on the cell membrane may take part in the transmission of EGCg signalling to protect cardiac cells from oxidative stress. Using an in vitro H_2_O_2_-induced oxidative stress model in H9c2 cells and an in vivo rat model of myocardial ischemia, we demonstrated the involvement of Cav in GTPs-mediated cardioprotection. In addition, we showed that EGCg-mediated Cav-1 activation could be modulated by Akt/GSK-3β signalling in H_2_O_2_-induced H9c2 cell injury. Taken together, our data suggest that EGCg may act to protect cardiac cells from H_2_O_2_-induced oxidative stress through Akt/GSK-3β dependent Cav signalling pathway.

## Methods

### Chemicals and reagents

H9c2 cell lines were purchased from American Type Culture Collection (ATCC, CRL-1446) (Rockville, MD). All reagents used were ACS or MB grade. EGCg, purchased from Sigma, was prepared as a stock solution of 10 mM by dissolving the compound in deionized water.

### Cell culture, EGCg and/or H_2_O_2_ treatments, MTT assay

H9c2 cells were cultured in Dulbecco’s modified essential medium (DMEM, Gibco, Invitrogen Taiwan Ltd., Taipei, Taiwan) containing 10% fetal bovine serum (FBS) (Gibco), 25 mM D-glucose, 2 mM L-glutamine, 1 mM sodium pyruvate, 1% streptomycin (100 μg/ml) (Gibco), and 1% penicillin (100 U/ml) (Gibco) at pH 7.4 in a 5% CO_2_ incubator at 37°C. Cell viability was measured using the MTT (3-(4,5-dimethylthiazol-2-yl)-2,5-diphenyltetrazolium bromide) cell proliferation assay (ATCC, Manassas, VA, USA). Cells (10^5^) were seeded onto 6-cm plates in DMEM-10% FBS. After adhering overnight, the cells were changed to serum-free medium with or without EGCg for 30 min in a 5% CO_2_ incubator at 37°C and then washed with phosphate buffer solution (PBS). The washed cells were treated with different concentrations of H_2_O_2_ in serum-free DMEM for 30 min in a 5% CO_2_ incubator at 37°C. After washing with PBS, the cells were incubated in serum-free DMEM for 24 h in a 5% CO_2_ incubator at 37°C. After 24 h incubation, MTT was then added to the cells at a final concentration of 0.5 mg/ml and the mixture was incubated at 37°C for 4 h. The optical density of the purple MTT formazan product was measured at 570 nm using a microplate reader (Anthos 2000, Austria).

### Determination of cellular Ca^2+^ levels

Fura 2-AM (fura 2-tetra-acetoxymethyl ester; Molecular Probes, Eugene, OR) was used as the fluorescent indicator. H9c2 cells were dissolved in PBS containing 2 mM fura 2-AM and incubated for 45 min at room temperature and then for 30 min at 37°C, during which time the fura 2-AM was trapped inside by esterase cleavage. The cells were then washed twice with PBS and diluted to a density of 2 × 10^6^ cells/ml in PBS. Recordings were made in a Perkin-Elmer LS 50B spectrofluorimeter equipped with an accessory to measure Ca^2+^ (Beaconsfield, Buckinghamshire, England). The dye trapped inside the cells was excited every second by exposure to alternating 340 and 380 nm light beams and the intensity of light emission at 510 nm was measured, allowing the monitoring of both the light intensity and the 340 nm fluorescence/380 nm ratio (F340/F380). The 340/380 ratio (R) was calculated and converted to the corresponding levels of [Ca^2+^]_i_ as described previously [20], using a Kd of 0.14 μM [21]:

$${\left[{\mathrm{Ca}}^{2+}\right]}_{i}\;=\;\mathrm{Kd}\;*\;\left(R‒\mathrm{Rmin}\right)/\left(\mathrm{Rmax}‒R\right)\;*\;{\mathrm{Sf}}_{2}/{\mathrm{Sb}}_{2}$$

where Rmin and Rmax are the ratios measured by the release of intracellular dye with 2 mM EGTA in 0.1% Triton X-100 (R_min_) followed by the addition of 2.1 mM Ca^2+^ (R_max_), whereas Sf_2_/Sb_2_ is the ratio of the 380 nm signals in Ca^2+^-free and Ca^2+^-replete solutions, respectively.

### Measurement of intracellular ROS generation by fluorescence spectrophotometry

Intracellular ROS levels were assessed using 2′,7′-dichlorofluorescein diacetate (DCF-DA) [21]. Cells (1.2 × 10^6^) loaded with DCF-DA in 3 ml PBS at a final concentration of 10 μM were incubated at 37°C for 1 h. After incubation, the cells were then washed three times with PBS by centrifugation at 300 × g at 4°C for 5 min. The cells re-suspended with PBS and brought to a density of 10^5^ cells/ml were measured for DCF-DA fluorescence changes every 10 min after the addition of H_2_O_2_ or EGCg by fluorescence spectrophotometry. The fluorescence excitation maximum for DCF-DA was 495 nm, and the corresponding emission maximum was 527 nm.

### Cell cycle phase determination

H9c2 cells (10^7^) were seeded in a 10-cm dish in DMEM-0.2% FBS and cultured in a CO_2_ incubator at 37°C for 24 hr. The cells were then changed to fresh medium, trypsinized, and centrifuged. The pellet was washed and re-suspended in 1 ml of pre-chilled PBS, fixed by the gradual addition of 3 ml of 95% ethanol, and stored in a deep freezer (-20°C) overnight. The cells were then washed three times by centrifugation and re-suspended in pre-chilled PBS. To stain the cells with propidium iodide (PI), the cells were re-suspended in PBS containing 0.1% Triton X-100, 20 μg/ml of PI, and 0.2 mg/ml of RNase A and incubated for 30 min at room temperature in the dark. Samples were analyzed on a flow cytometer (FC500 Flow Cytometry System, Beckman Coulter, Inc.) with a 488 nm excitation laser. The cell cycle phases were determined using the software provided with the instrument (CXP Software, Beckman Coulter, Inc.) [22].

### Western blots

The sample preparation for SDS-PAGE and electro-transfer was as described previously [23,24]. The primary antibodies used were mouse monoclonal antibodies against β-actin (C4) (sc-47778), human N-cadherin (H-63) (sc-7939), human-β-catenin (9 F2) (sc-47752), human GSK-3β (H-76) (sc-9166), human pGSK-3β (pY-216) (sc-135653), human cyclin D1 (DSC-6) (sc-20044), Cav-3 (A-3) (sc-5310), rat-nCx43 (D-7) (sc-13558), and rabbit GAPDH (6C5) (sc-32233) (all from Santa Cruz and all 1: 1000 dilution); goat polyclonal anti-human Laminin-R antibody (F-18) (sc-21534) (Santa Cruz; 1: 1000 dilution); and rabbit polyclonal antibodies raised against human Cav-1 (N-20) (sc-894), human Akt1 (H-136) (sc-8312), human Ser 9 phosphorylated GSK-3β (Ser 9) (sc-11757), pCav-1 (Tyr 14) (sc-101653) (Santa Cruz; 1: 1000 dilution), human Ser 473 phosphorylated Akt1 (SAB4504331) (Sigma, 1: 500 dilution), and rat Cx43 (71-0700) (Zymed, Invitrogen; 1: 1000 dilution). After 3 × 10 min washes with PBS containing 0.05% Tween-20, the membrane was incubated for 2 h at 4°C with alkaline phosphatase-conjugated goat anti-rabbit, donkey-anti-goat, or rabbit-anti-mouse IgG antibodies (Santa Cruz; 1:5000 dilution), and the bound antibody was detected using 5-bromo-4-chloro-3-indolyl phosphate-nitro blue tetrazolium.

### EGFP-expressing H9c2 and fluorescence measurements

EGFP-expressing H9c2 cells were generated by co-transfecting pEGFP-N1 (PT3027-5) vector with Lipofectamine 2000 (Invitrogen) into H9c2 cells. The fluorescence changes in transformed cells were measured in a Perkin-Elmer LS 50B spectrofluorimeter (Beaconsfield, Buckinghamshire, England). The fluorescence excitation maximum for EGFP was 488 nm, and the corresponding emission maximum was 507 nm [20].

### Immunoprecipitation and immunoblotting

EGFP expressed H9c2 cells were lysed with pre-chilled RIPA buffer containing 50 mM Tris–HCl, pH 7.4, 150 mM NaCl, 1% Nonidet P-40, 0.25% sodium deoxycholate, 5 mM EDTA, 0.02 mM EGTA, 1% phenylmethanesulfonyl fluoride, and a cocktail of protease inhibitors. The cell lysates were diluted with pre-chilled PBS to a volume of 500 μl and a concentration of 5 mg/ml and incubated overnight at 4°C with 25 μg of rabbit anti-EGFP (PG-10013, Genesis Biotech Inc., Taipei, Taiwan). 50 μl protein G Sepharose 4 Fast flow (GE Healthcare UK Ltd., England) was then added, and the mixture was incubated for 1 h at 4°C. After centrifugation, the pellet was washed with RIPA buffer followed by Tris-OH buffer (50 mM, pH 8.0). The samples dissolved in reducing buffer containing 1% SDS, 100 mM dithiothreitol, 50 mM Tris-OH, pH 7.5 were used for molecular identification of the protein complexes that formed with EGFP in the overexpressed cells by SDS-PAGE, followed by immunoblotting, as described above. In addition, protein bands on the SDS-PAGE gels were cut out for molecular identification by acquiring MALDI-MS spectra at the Proteomics center at National Chung-Hsing University (Taichung, Taiwan) (Additional file 1: Figure S1).

### Protein separation by 2-DE and isoelectric focusing (IEF)

After co-immunoprecipitation, the protein complexes conjugated with EGFP were separated by two-dimensional electrophoresis (2-DE) and IEF. Immobilized pH gradient strips (pH 3-10, 13 cm) were rehydrated with 450 μg protein at room temperature overnight (at least 12 h). IEF was performed using an IPGphor 3 apparatus (GE healthcare) for a total of 17 kVh at 20°C. After IEF, strips were equilibrated in 6 M urea, 75 mM Tris–HCl (pH 8.8), 29.3% (v/v) glycerol, 2% (w/v) SDS and 0.002% (w/v) bromophenol blue with 65 mM DTT for 15 min and in the same buffer with 240 mM iodoacetamide for next 15 min. Strips were then transferred onto 10% polyacrylamide gels and sealed with 0.5% (w/v) low-melting-point agarose in SDS running buffer containing 0.02% (w/v) bromophenol blue. The gels were run in a PROTEAN® II xi gel tank (Bio-Rad) at 35 mA per gel at 20°C until the dye reached the bottom of the gels. Gels were stained with Bio-safe™ Coomassie G-250 Stain (Bio-Rad) according to the manufacturer’s protocol. Stained gels were scanned using Scanmaker 9800XL (Microtek) and analyzed using ImageMaster™ 2D Platinum 7.0 (GE healthcare). The proteins of interest were cut out for molecular identification by acquiring MALDI-MS spectra.

### RNA extraction and semi-quantitative RT-PCR

The procedures for RNA extraction and semi-quantitative reverse transcription polymerization chain reaction (semi-quantitative RT-PCR) have been described previously [20]. The primers used were GAPDH, (forward) 5-ACC ACA GTC CAT GCC ATC AC-3, (reverse) 5-TCC ACC ACC CTG TTG CTG TA-3, product size 452 bp; Cav-1, (forward) 5-CTA CAA GCC CAA CAA CAA GGC-3, (reverse) 5-AGG AAG CTC TTG ATG CAC GGT-3, product size 342 bp; Cav-2, (forward) 5-GCT CAA CTC GCA TCT CAA GCT-3, (reverse) 5-TCT GTC ACA CTC TTC CAT ATT-3, product size 260 bp; Cav-3, (forward) 5-GGA CAT TGT GAA GGT GGA TTT-3, (reverse) 5-GCA CTG GAT CTC AAT CAG GTA-3, product size 247 bp. The correct sequences for all genes were confirmed by alignment with the reported sequence for each gene.

### A rat model of myocardial ischemia involving LAD ligation

Male Sprague–Dawley rats (200-250 g), aged 8-9 weeks, were randomly divided into three groups: a sham control group, a group that underwent LAD ligation without GTP supplementation, and a group that underwent LAD ligation with GTP supplementation (400 mg/Kg/day) for 2 weeks, with 5 animals per group. Once the rat was anesthetized, the heart was exposed via a left thoracotomy, and a 6-0 polypropylene suture was tied onto the LAD coronary artery 3 mm distal to the inferior margin of the left atrium, and the chest wall was closed in layers. Regional myocardial ischemia was confirmed by the observation of a rapid change from reddish to a dark red color on the anterior surface of the LV and rapid development of akinesia and dilatation in the ligated area. All experimental procedures conformed to the “Guidelines for Proper Conduct of Animal Experiments” approved by the Animal Care and Use Committee of Taichung Veterans General Hospital and National Chung-Hsing University.

After surgery, the rats were fed intragastrically with GTPs (400 mg/kg) every day for two weeks. After the rats were sacrificed, the hearts were cut along the long cross-sectional axis of the left ventricle, and cardiac tissues at both the infarcted area and a remote site of myocardium were isolated to determine protein levels of the 67 kD laminin receptor and Cav-1 and-3 by immunoblotting, as described above.

### Statistical analysis

Quantitative values are presented as the mean and standard error of the mean (mean ± SEM). A difference was considered to be statistically significant when the P value was less than 0.05.

## Results

### EGCg cardioprotective effects on cell viability, ROS formation, and cytosolic Ca^2+^ overload in H_2_O_2_-treated H9c2 cells

**Figure 1:**
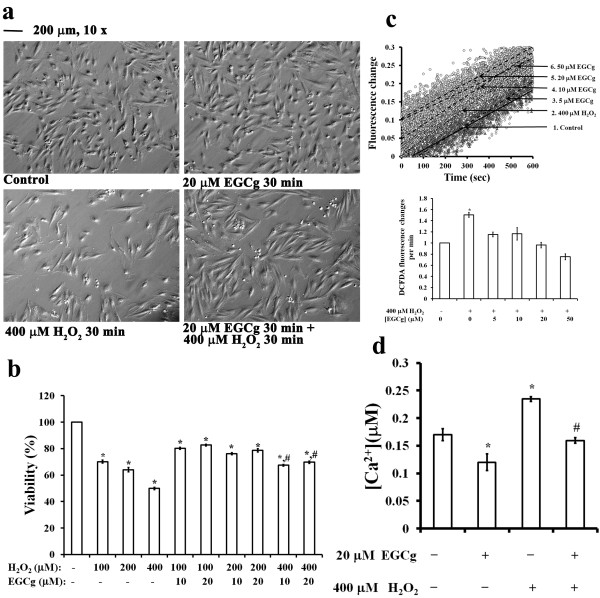
**A cell model illustrating cardioprotection of EGCg on H**_**2**_**O**_**2**_**-induced oxidative stress in H9c2 cells. (a)** Phase contrast microscopy showing cell morphology of H9c2 cells in the conditions of control (left top), 20 μM EGCg treatment for 30 min (right top), 400 μM H_2_O_2_ exposure for 30 min (left bottom), and 20 μM EGCg pre-treatment for 30 min followed by 400 μM H_2_O_2_ exposure for 30 min (right bottom). Calibration bar of 200 μm as indicated. **(b)** MTT assay of cell viability after incubation with 0, 100, 200, and 400 μM H_2_O_2_ with or without 0, 10, and 20 μM EGCg for 30 min. **(c)** Measurements of intracellular ROS formation by DCF-DA in H9c2 cells. The fluorescence changes of DCF-DA-loaded cells were measured every 10 min before and after the addition of 400 μM H_2_O_2_ with 0-50 μM EGCg as indicated by fluorescence spectrophotometry. The fluorescence excitation maximum for DCF-DA was 495 nm, and the corresponding emission maximum was 527 nm. **(d)** Effects of H_2_O_2_ and/or EGCg on intracellular Ca^2+^ levels in H9c2 cells. Cellular Ca^2+^ levels were measured using the Fura-2 fluorescence ratio (F340/F380) in H9c2 cells cultured in the conditions of control, 20 μM ECGg treatment for 30 min, and 400 μM H_2_O_2_ exposure for 30 min with and/or without 20 μM ECGg treatment for 30 min, then during the measurement in PBS for 3 min periods. The F340/F380 ratio was continuously monitored. In **b, c** and **d**, the values are the mean ± SEM (n = 6), with *, # indicating a significant difference compared to the untreated cells or the H_2_O_2_-treated cells, respectively.

Previously, we have demonstrated that pre-treatment with green tea extract protects cardiomyocytes from regional myocardial ischemia by overcoming cytosolic Ca^2+^ overload, myofibril disruption, and alterations in adherens and gap junction protein levels and distribution in rats [4]. In the present study, we used a cell model of H9c2 rat cardiomyoblast to verify the cardioprotection of EGCg against the H_2_O_2_-induced oxidative stress during myocardial ischemia assault. When exposed to 400 μM H_2_O_2_, an increase in oxidative stress caused morphological changes in H9c2 cells, which were accompanied by an increase in cell death that was prevented by 20 μM EGCg pre-treatment for 30 min (Figure 1a). Note that EGCg alone did not significantly alter cell morphology (Figure 1a). In addition, the MTT assay showed a dose-dependent decrease in cell viability of H9c2 cells treated with H_2_O_2_ from 100 to 400 μM (Figure 1b). EGCg pre-treatment with 10 or 20 μM for 30 min did not improve viability in cells treated with 100 or 200 μM H_2_O_2_, but a 25% recovery of cell viability was observed after exposure to 400 μM H_2_O_2_ (Figure 1b). Measurements of intracellular ROS formation in H9c2 cells demonstrated that 5 to 50 μM EGCg attenuated 30% ROS formation in H_2_O_2_-treated cells (Figure 1c). Measurements of the fura-2 F340/F380 fluorescence ratio of these cells also indicated that EGCg could attenuate the cytosolic Ca^2+^ in H9c2 cells with or without H_2_O_2_ exposure (Figure 1d). The cellular Ca^2+^ concentrations (μM) for the cells cultured were 0.17 ± 0.01 in the control medium, 0.12 ± 0.01 in the medium containing 20 μM EGCg for 30 min, 0.23 ± 0.004 in the medium containing 400 μM H_2_O_2_ for 30 min, and 0.16 ± 0.004 in the condition of 400 μM H_2_O_2_ exposure for 30 min followed by 20 μM EGCg treatment for 30 min, respectively (Figure 1d).

### Effects of EGCg and H_2_O_2_ on the protein levels of N-cadherin, β-catenin, and phosphorylated and non-phosphorylated Cx43 in H9c2 cells

**Figure 2:**
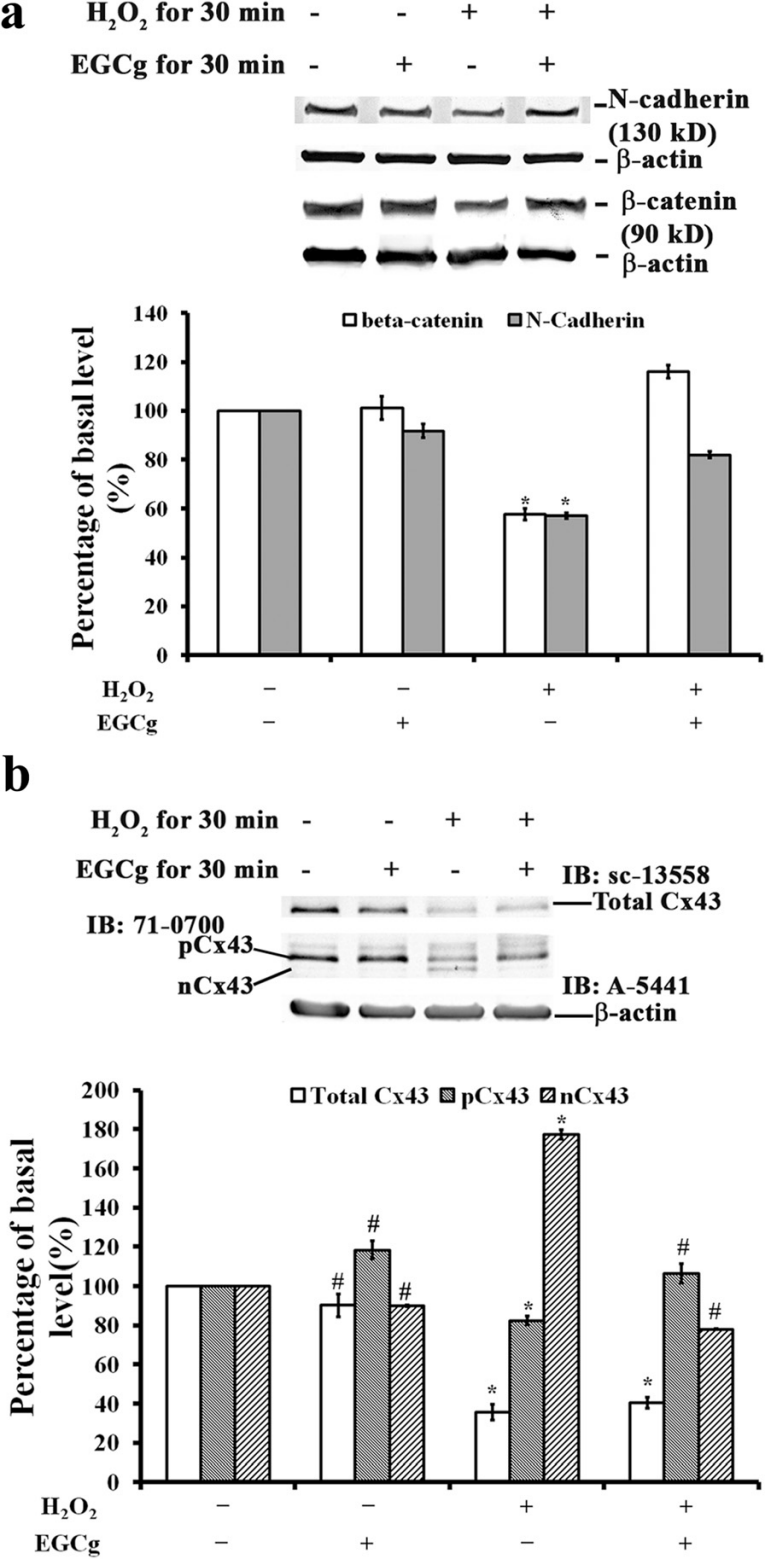
**Effects on the levels of β-catenin, N-cadherin, and Cx 43 in H9c2 cells. (a)** Western blotting (top) with quantitative analyses (bottom) of N-Cadherin, β-catenin, and β-actin levels in the H9c2 cells of controls with (lane 2) or without (lane 1) 20 μM EGCg pre-treatment for 30 min or 400 μM H_2_O_2_-induced oxidative stress for 30 min with (lane 4) or without (lane 3) 20 μM EGCg pre-treatment. **(b)** Top: Cx43 levels on western blots using mouse monoclonal anti-rat Cx43 antibody (sc-13558) or rabbit polyclonal antibodies (71-0700) in the H9c2 cells of controls with (lane 2) or without (lane 1) 20 μM EGCg pre-treatment for 30 min or 400 μM H_2_O_2_-induced oxidative stress for 30 min with (lane 4) or without (lane 3) 20 μM EGCg pre-treatment. Bottom: Quantitative analyses using β-actin as the loading control. In **a** and **b**, the values are the mean ± SEM (n = 6), with *, # indicating a significant difference compared to the untreated cells or the H_2_O_2_-treated cells, respectively.

To determine whether EGCg has a protective effect on changes in adherens and gap junction proteins in H_2_O_2_-treated H9c2 cells, we examined the effect of EGCg on differential expression of the adhesion molecules N-cadherin and β-catenin, and the gap junction protein Cx43 in H_2_O_2_-treated H9c2 cells (Figure 2). Western blot analysis revealed a decrease in N-cadherin (42.9%) and β-catenin (42.3%) protein content in cells exposed to H_2_O_2_ for 30 min compared to controls, but not in EGCg-pre-treated cells with or without H_2_O_2_ (Figure 2a). To measure levels of phosphorylated and non-phosphorylated Cx43 in cells, two different antibodies were used. Mouse monoclonal anti-rat-Cx43 antibody labelled one band with a molecular weight of 43 kD (Figure 2b), and the intensity of this band was reduced in H_2_O_2_-exposed cells with (59%) or without (64%) EGCg pre-treatment compared to controls (Figure 2b). The rabbit polyclonal anti-Cx43 antibody is known to recognize both phosphorylated (pCx43, 43 kD) and non-phosphorylated (nCx43, 41 kD) Cx43 isoforms on polyacrylamide gels [4,25]. The intensity of phosphorylated pCx43 was reduced in H_2_O_2_-treated cells without EGCg pre-treatment (17% decrease) but not changed with EGCg pre-treatment compared to controls (Figure 2b). In contrast, the intensity of non-phosphorylated nCx43 was increased in H_2_O_2_-treated cells without EGCg pre-treatment (77%) but decreased with EGCg pre-treatment (22%) compared to controls (Figure 2b). This result suggests that the H_2_O_2_-induced oxidative stress might cause destruction of gap junction formation by increasing the levels of non-phosphorylated nCx43 in cardiac cells, while EGCg pre-treatment could attenuate such damage on the gap junction assembly in H_2_O_2_-treated cells.

### Effects of EGCg and H_2_O_2_ on the cell cycle and phosphorylated and GSK-3β, β-catenin, and cyclin D1 protein levels in H9c2 cells

**Figure 3:**
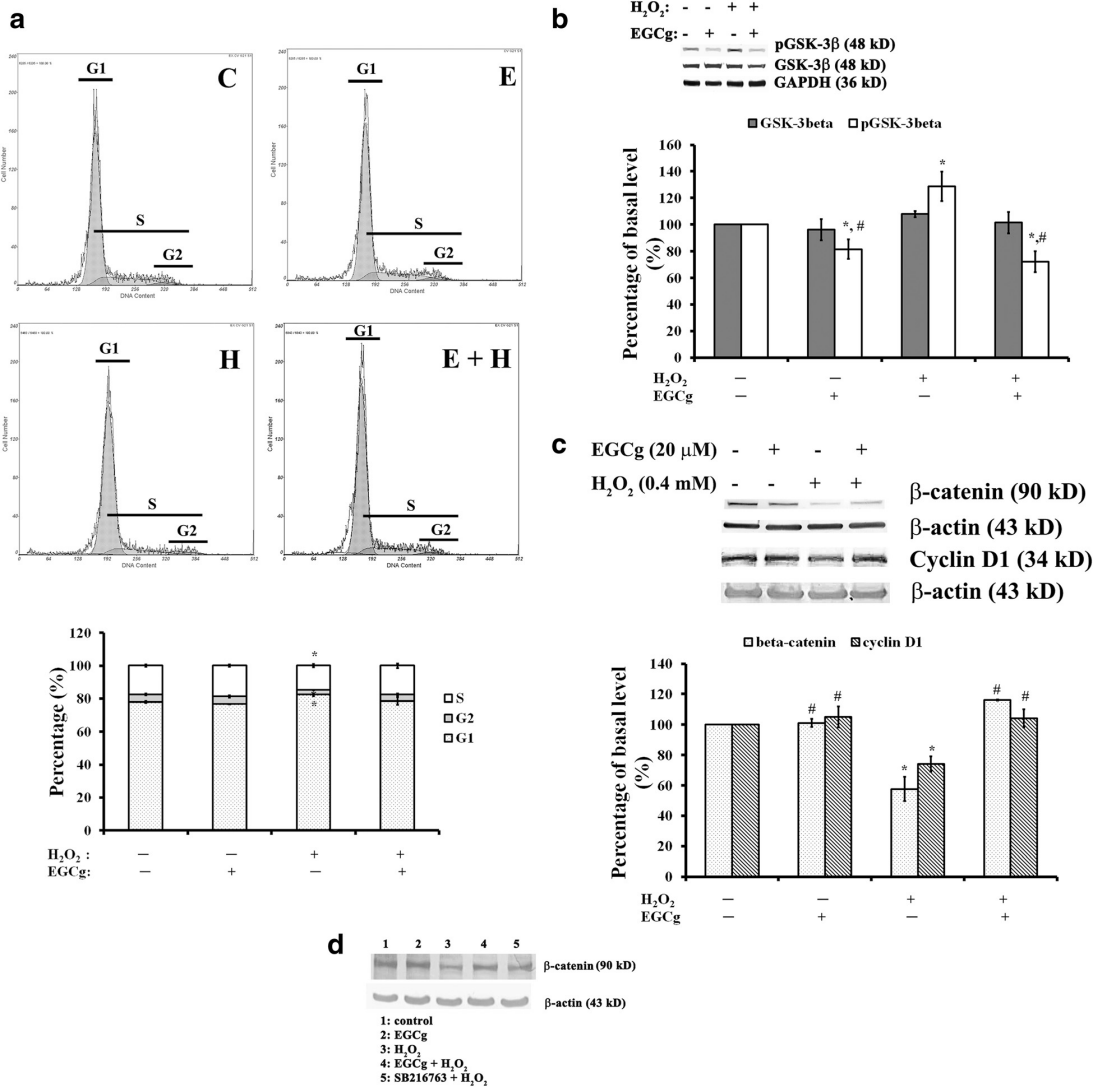
**Effects on the cell cycle, and phosphorylated GSK-3β, GSK-3β, β-catenin, and cyclin D1 in H9c2 cells. (a)** Top: Cell cycle phase determined by flow cytometry for H9c2 cells in the control medium (left top, label C), or in the medium containing 20 μM EGCg for 30 min (right top, label E), or in the medium containing 400 μM H_2_O_2_ for 30 min with (right bottom, label E + H) or without 20 μM EGCg pretreatment for 30 min (left bottom, label H). Bottom: Quantitative analysis of the cell cycle phase. **(b)** Western blotting (top) with quantitative analyses (bottom) of pGSK-3β, GSK-3β, and GAPDH levels for the H9c2 cells cultured in the medium as indicated. **(c)** Western blotting (top) with quantitative analyses (bottom) of β-catenin, cyclin D1, and β-actin levels for the H9c2 cells in the medium with the addition as indicated. **(d)** Western blots showing inhibition of GSK-3β on β-catenin levels in the H9c2 cells. Lane 1: cells cultured in the control medium; lane 2: cells cultured in the medium containing 20 μM EGCg for 30 min; lane 3: cells cultured in the medium containing 400 μM H_2_O_2_ for 30 min, lane 4: cells cultured in the medium containing 400 μM H_2_O_2_ for 30 min with 20 μM EGCg pre-treatment for 30 min, lane 5: cells cultured in the medium containing 400 μM H_2_O_2_ for 30 min with the pretreatment of 10 μM SB 216763 inhibitor of GSK-3α/3β for 30 min. In **a, b,** and **c**, the values are the mean ± SEM (n = 6), with *, # indicating a significant difference compared to the cells in control medium and the cells treated with H_2_O_2_, respectively.

Flow cytometry revealed that the incubation with 400 μM H_2_O_2_ for 30 min blocked DNA synthesis and G1 entry into the S phase of the cell cycle in H9c2 cells (Figure 3a). After H_2_O_2_ treatment, the number of cells in G0/G1 phase increased by 5%, but the number in S phase and in G2 phase decreased by 16% and 36%, respectively, compared to controls. The corresponding values for EGCg treatment with or without H_2_O_2_ were not significantly different from controls (Figure 3a).

Glycogen synthase kinase 3β (GSK-3β) is a key component of multiple signalling pathways involved in the regulation of cell fate, protein synthesis, glycogen metabolism, cell mobility, proliferation, and survival [26-28]. By preventing cells from entering the cell cycle, GSK-3β participates in the regulation of the β-catenin signalling pathway by modulating cyclin D1 expression levels [29]. For cells treated with 400 μM H_2_O_2_, phosphorylation of GSK-3β (Tyr216) was increased by 20%, whereas EGCg pre-treatment with or without H_2_O_2_ attenuated both total and phosphorylated GSK-3β levels in cells (Figure 3b). In addition, H_2_O_2_ decreased β-catenin (42%) and cyclin D1 (26%) expression levels (Figure 3c), which may cause the subsequent cell cycle arrest at the G1-S phase (Figure 3a). This H_2_O_2_-induced inhibition of the β-catenin/cyclin D1 signalling pathway could be efficiently prevented by pre-treatment with 20 μM of EGCg (Figure 3c) or ~10 μM of SB 216763, an inhibitor of GSK-3α/3β (Figure 3d).

### EGCg-induced fluorescence changes in intact Triton X-100-soluble and insoluble fractions of EGFP-expressing H9c2 cells

**Figure 4:**
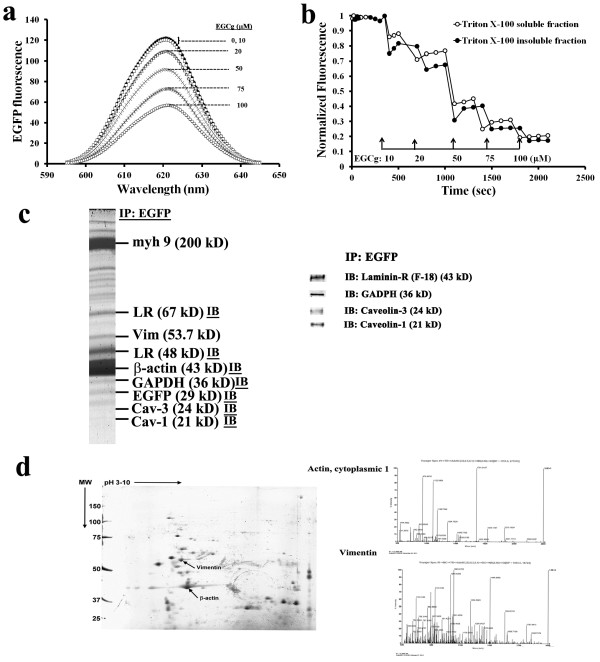
**EGCg-induced fluorescence changes in EGFP-expressing H9c2 cells and molecular identification on the EGFP-conjugated protein complex. (a)** Fluorescence spectra showing the dose effect of EGCg on EGFP fluorescence. **(b)** Normalized EGFP fluorescence in the absence or presence of 0.1% Triton X-100. Fluorescence spectroscopy was performed as described in the Materials and Methods. The fluorescence emitted at 507 nm measured in the absence of EGCg was used to normalize the fluorescence changes caused by EGCg titrations. Each value is the mean of six measurements. **(c)** The EGFP co-precipitated proteins were separated by one-dimensional SDS-PAGE or **(d)** two-dimensional electrophoresis (2-DE), followed by proteomics acquiring MALDI-MS spectra. A co-immunoprecipitation assay reveals molecular identities by immunoblotting (IB) of the protein complexes (i.e., LR, β-actin, GAPDH, Cav-1 and -3) formed with EGFP in these cells.

To investigate the role of EGCg-mediated transmembrane signalling in cardioprotection, EGFP was ectopically expressed in H9c2 cells. Fluorescence spectroscopy indicated that increases in EGCg concentrations from 0 to 100 μM caused dose-dependent decreases in EGFP fluorescence (Figure 4a). In addition, this experimental approach allowed us to monitor the fluorescence changes as a means to distinguish the effects of Triton X-100-soluble and insoluble compartments on cell membrane (Figure 4b).

To identify the protein complexes conjugated to EGFP in EGFP-expressing cells, a co-immunoprecipitation assay using a specific antibody against EGFP was used for molecular identification, followed by immunoblotting or MS spectra. The EGFP co-immunoprecipitated proteins separated by one-dimensional SDS-PAGE (Figure 4c, Additional file 1: Figure S1) or 2-D electrophoresis (Figure 4d) were myosin IX [myh 9, 200 kD, EDM15905], 67 kD laminin receptor [LR, 67 kD, 48 kD], vimentin [53.7 kD, NP112402], β-actin [43 kD, ABM16832)], GAPDH (36 kD), Cav-3 (24 kD) and Cav-1 (21 kD). LR has been identified as a receptor for EGCg [30]. Cav-1 and -3 are known to stabilize lipid raft microdomains (Triton X-100-resistant compartment) on cell membranes [31,32]. Cytoskeletal proteins including β-actin, myosin IX, and vimentin may form complexes with other protein in cellular Triton X-100-resistant microdomains, which may play a role in EGCg transmembrane signalling in cardiac cells.

### Effects of H_2_O_2_ and EGCg on the expression of Cavs in H9c2 cells

**Figure 5:**
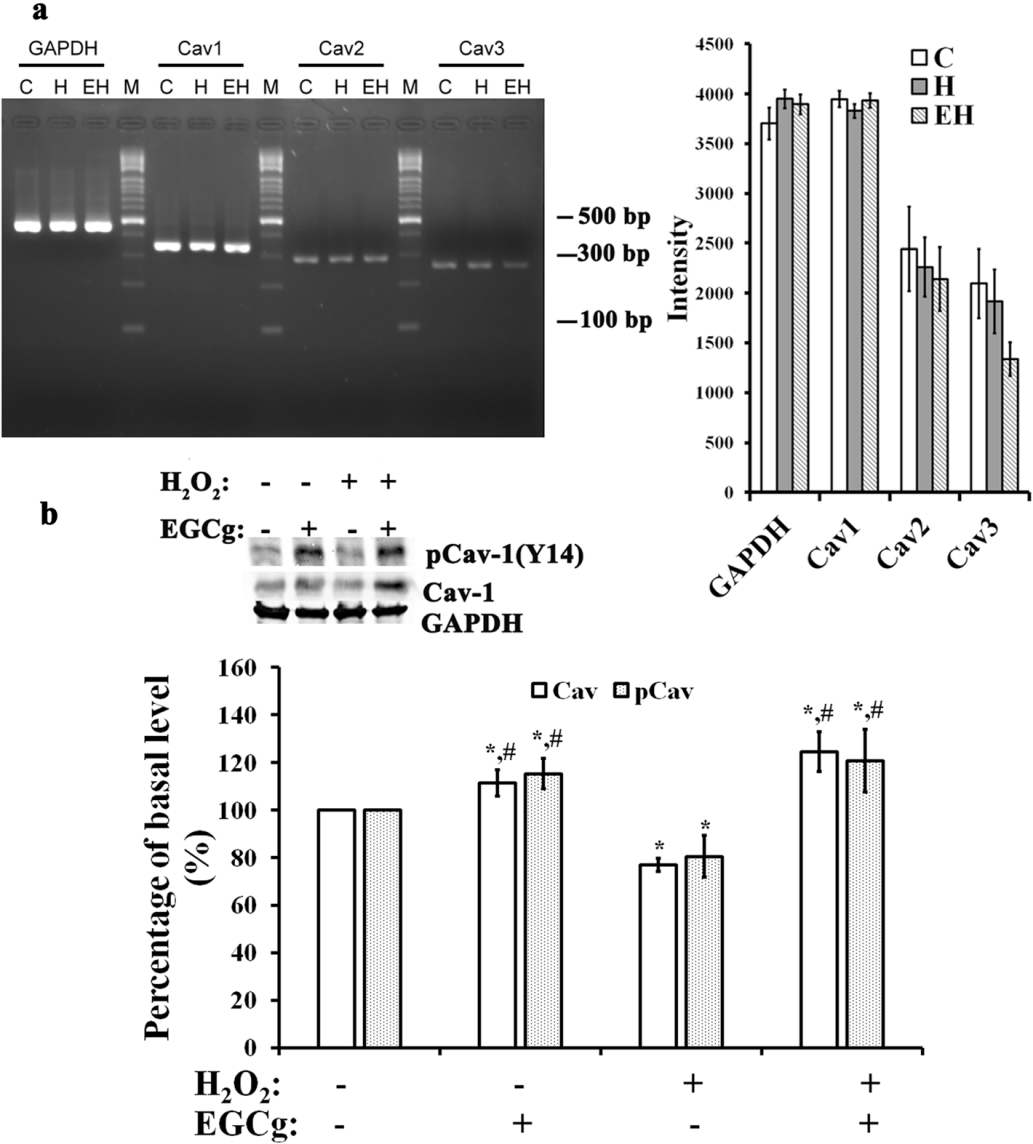
**Effects of H**_**2**_**O**_**2 **_**and/or EGCg on the expression of Cav in H9c2 cells. (a)** Agarose gels demonstrating the presence of mRNAs encoding different Cav isoforms (Cav-1, Cav-2, and Cav-3) (left), and a histogram showing the Cav isoform mRNA levels relative to those of GAPDH mRNA in cells exposed to 0.4 mM H_2_O_2_ with (EH) or without 20 μM EGCg pretreatment (H) (right). **(b)** Immunoblot analysis reporting the protein levels of Cav-1 and phosphorylated Cav-1in whole cell lysates of H9c2 cells cultured in the medium as indicated. GAPDH was used as the internal control for data analysis. In **a** and **b**, each value is the mean ± SEM (n = 6). *indicates significant difference compared to H9c2 cells in control condition (C), and # symbolizes a significant difference compared to cells treated with H_2_O_2_ (H).

H9c2 cells expressed mRNA encoding Cav-1, Cav-2 and Cav-3, with a dominant Cav-1 expression (Figure 5a). Exposure to 400 μM H_2_O_2_ with (EH) or without 20 μM EGCg pre-treatment (H) did not show significant effects on mRNA expression for any isoform as compared to controls. Western blot analysis indicated that protein levels of Cav-3 in whole-cell lysates were not significantly changed by H_2_O_2_ and/or EGCg (data not shown). H_2_O_2_ induced a 30% decrease in the levels of Cav-1 concomitant with a 20% decrease in phosphorylated Cav-1. These decreases were abrogated by pre-treatment with 10 or 20 μM EGCg for 30 min (Figure 5b). For cells with EGCg treatment for 30 min, the levels of Cav-1 and phosphorylated Cav-1 were increased by 12% and 15%, respectively.

### Effects of LAD ligation and GTP treatment on the protein content of LR and Cav-1 and -3 in rat myocardium

**Figure 6:**
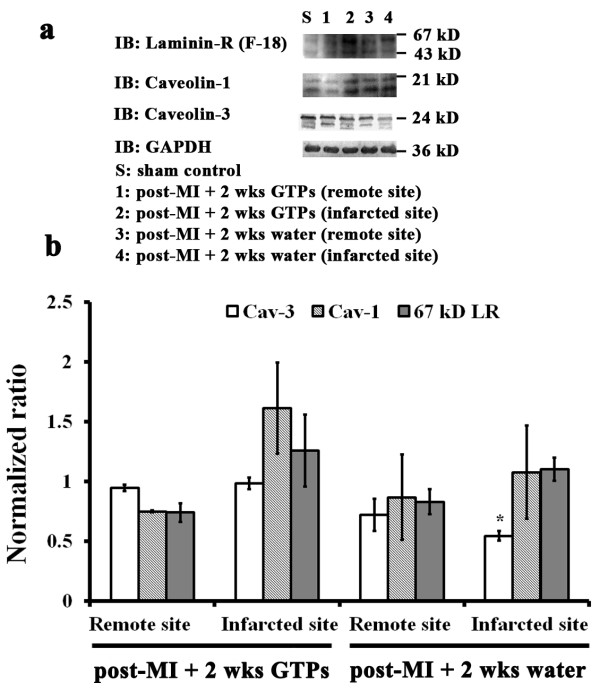
**Effects of LAD ligation and GTPs on the myocardial content of LR, Cav-1, and Cav-3. (a)** Western blotting with **(b)** quantitative analyses of 67 kD laminin receptor, Cav-1, and Cav-3 in the myocardium of sham controls (lane S) or post-LAD ligated rats with GTP supplementation (lanes 1, 2) or with water (lanes 3, 4) for 14 days. For post-LAD ligated rats supplemented with GTPs or water, cardiac tissues at the infarcted area (lanes 2, 4) and a remote myocardial site (lanes 1, 3) were isolated for analysis. Each group contains 5 animals for data analyses. The values are the means ± SEM, with *indicating a significant difference compared to the sham controls.

Using a rat model of LAD ligation-induced myocardial ischemia, we demonstrated the effects of GTP treatment for 2 weeks on the expression of LR and Cav-1 and -3 in the myocardium (Figure 6). Two bands with molecular weights of 43 and 67 kD were labelled for LR and at 21 kD for Cav-1. The band intensities for LR and Cav-1 were not significantly different in sham controls and post-LAD ligation with or without 2-week GTP treatment. In contrast, one major band appeared at 24 kD for Cav-3, which was significantly reduced in infarcted myocardium (46%) but not significantly different in remote myocardium after ligation without GTPs compared to sham controls. In post-LAD-ligated rats treated with GTPs for 2 weeks, the band intensity for Cav-3 in both infarcted and remote myocardium was similar to sham controls. This result suggests that the expression of Cav-3 is involved in signalling events for GTP-mediated cardioprotection against myocardial ischemic injury.

### Effects of H_2_O_2_ and EGCg on the Akt/GSK-3β survival pathway in H9c2 cells

**Figure 7:**
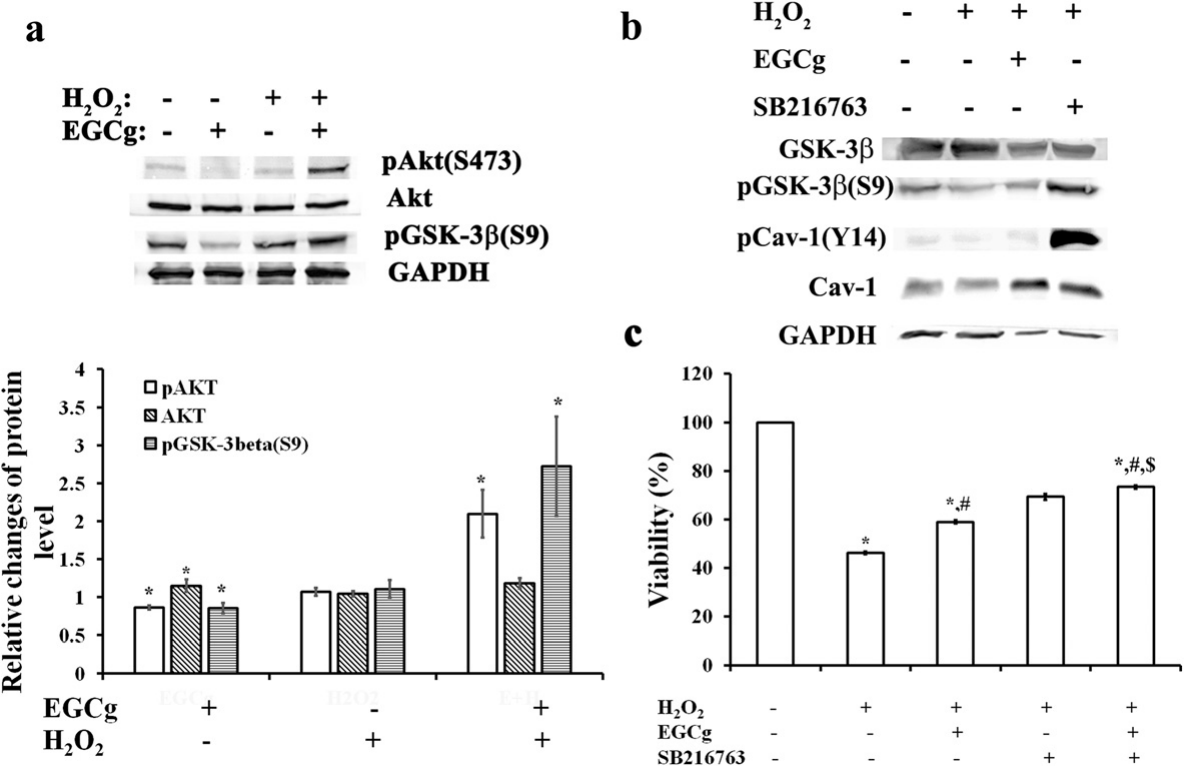
**The Akt pro-survival pathway associated with GSK-3β signalling takes part in EGCg-mediated Cav-1 activation. (a)** Western blotting with quantitative analyses of Akt phosphorylation at ser-473 and its downstream substrate GSK-3β phosphorylation at ser-9 in H9c2 cells. **(b)** Immunoblot analysis showing effects of EGCg and/or GSK-3β inhibition by GSK-3α/3β inhibitor, SB 216763, on the phosphorylation of pGSK-3β (S9) and pCav-1 (Y14) in H_2_O_2_-induced H9c2 cells. **(c)** MTT assay showing the improvement of H_2_O_2_,-suppressed cell viability by EGCg and/or GSK-3β inhibitor, SB 216763 pre-treatment in H_2_O_2_-induced H9c2 cells. In **a** and **b**, each value is the mean ± SEM (n = 6). *indicates significant difference compared to H9c2 cells in control condition, and # symbolizes a significant difference compared to cells treated with H_2_O_2_ (H). In **a** and **c**, each value is the mean ± SEM (n = 6). *indicates significant difference compared to H9c2 cells in control condition, # symbolizes a significant difference compared to cells treated with H_2_O_2_, and $ represents a significant difference compared to cells pretreated with EGCg in prior to H_2_O_2_ treatment.

Myocardial Akt signalling pathway is known to play an important role in the regulation of many cellular functions including growth, survival, proliferation, metabolism, glucose uptake, gene expression, and cell-cell communication [33]. To examine whether the Akt pro-survival pathway associated with GSK-3β signalling takes part in EGCg-mediated cardoioprotection in an H_2_O_2_-induced H9c2 cardiomyoblast injury, we determined effects of H_2_O_2_ and EGCg on the Akt phosphorylation at ser-473 and its downstream substrate GSK-3β phosphorylation at ser-9 in H9c2 cells by western blot analysis (Figure 7a). Treatment with 20 μM EGCg for 30 min decreased 14% pAkt (S473) in concomitant with 15% increase of total Akt and 15% decrease of pGSK-3β (S9) in H9c2 cells. Incubation with 400 μM H_2_O_2_ alone for 30 min did not show significant effects on the level for pAkt (S473), total Akt, and pGSK-3β (S9) in cells. However, for cells pre-treated with EGCg for 30 min in prior to H_2_O_2_ exposure, the levels of pAkt (S473), total Akt, and pGSK-3β (S9) were increased by 2.1 folds, 18% and 2.7 folds, respectively. This suggested that cellular survival induced by EGCg converges on Akt activation such as to blockade of GSK-3β activity and initiation of protective signalling events in H_2_O_2_-induced H9c2 cells.

To further establish the relationship between Cav and GSK-3β signalling pathway, we determined the effects of GSK-3β inhibition on the phosphorylation of Cav-1 in H_2_O_2_-induced H9c2 cells (Figure 7b). For cells exposed to 400 μM H_2_O_2_, phosphorylation of pGSK-3β (S9) and pCav-1(Y14) was decreased, whereas EGCg or GSK-3β inhibitor, SB 216763 pre-treatment increased phosphorylation of both pGSK-3β (S9) and pCav-1(Y14) in H_2_O_2_-exposed cells (Figure 7b). Concomitantly, the H_2_O_2_,-suppressed cell viability of H9c2 was improved by EGCg and/or GSK-3β inhibitor, SB 216763 pre-treatment (Figure 7c) Apparently, EGCg mediated Cav-1 signalling through activation on Akt/GSK-3β might act to protect cardiac cells against the H_2_O_2_-induced oxidative stress in H9c2 cells.

## Discussion

Oxidative stress describing an imbalance between the generation and clearance of reactive oxygen species (ROS) in cells has the causative effect on the development and progression of heart disease [34,35]. A cell line of H9c2 rat cardiomyoblasts has been used as an in vitro cellular model for cardiac tissues in response to oxidative stress conditions [36]. In addition, H9c2 cells associated with H_2_O_2_-induced oxidative stress have been widely used to evaluate the protective role of EGCg against oxidative injury and cell death caused by ROS in cardiac cells [37]. In the present study, we demonstrated the cardioprotection effects of EGCg against H_2_O_2_-induced oxidative stress in H9c2 cells by preventing ROS formation and cytosolic Ca^2+^ overload (Figure 1). This is consistent with the finding by Dreger et al. [5], which demonstrated that EGCg treatment for 30 min significantly reduced intracellular levels of ROS in a model of H_2_O_2_-induced oxidative stress in neonatal rat cardiomyocytes.

Using the H9c2 cell model of H_2_O_2_-induced oxidative stress for a proteomics study, Chou et al. [36] showed that oxidative stress triggers tyrosine phosphorylation on target proteins associated with cell-cell junctions, the actin cytoskeleton, and cell adhesion in cardiac cells. Previously utilizing a surgical model of IR involving a temporary LAD ligation in rats, we demonstrated that green tea polyphenol (GTP) pre-treatment protects cardiomyocytes from IR injury by altering the expression and distribution of adherens and gap junction proteins [4]. In agreement with previous findings, the present study validated that EGCg has a protective effect on H_2_O_2_-induced changes in protein expression for the adherens molecules of β-catenin and N-cadherin and the gap junction protein Cx43 in H9c2 cells (Figure 2).

GSK-3β relevant to mitochondrial signalling has emerged as a key end-effector of multiple signalling pathways for cardioprotection [28]. Here, we demonstrated that EGCg pre-treatment could protect the H_2_O_2_-induced cell cycle arrest at the G1-S phase by decreasing tyr216 phosphorylation of GSK-3β, leading to the subsequent increase in β-catenin and cyclin D1 protein expression in H9c2 cells (Figure 3). β-catenin is a transcriptional activator of target genes in the nucleus [38,39]. Cyclin D1 is one of target genes that may be activated by β-catenin for cell proliferation [29]. EGCg modulation of the GSK-3β/β-catenin/cyclin D1 signalling pathway would therefore promote the cardiac cell cycle progression into S phase.

Many of the properties of lipid rafts have been inferred from detergent-resistant membranes that occur in non-ionic detergent (e.g., Triton X-100) lysates of cells [32]. In the present study, we determined the EGCg-induced fluorescence changes in intact, Triton X-100-soluble and insoluble fractions of these cells (Figure 4). Along with the molecular identification for the protein complexes with EGFP in these cells, these data suggested that the lipid raft microdomain-associated proteins (i.e., LR, Cav-1 and Cav-3) as well as cytoskeletal proteins (i.e., β-actin, myosin IX, and vimentin) may play a role in EGCg transmembrane signalling in cardiac cells. Both intact microtubules and actin filaments have been shown to be the primary interacting partners of lipid rafts [15,31,32]. There is increasing evidence that lipid rafts in the cell membrane are clustered in response to different stimuli to form signalling platforms for transmembrane transduction [40]. Among these signalling platforms, Zhang et al. [41] reported that some large redox signalling molecules are recruited into lipid raft microdomains and subsequently produce ROS in bovine coronary arterial endothelial cells. The present study comparing the binding of EGCg to EGFP-expressing cells in conditions with or without H_2_O_2_-induced oxidative stress indicated that the strength of EGCg binding to cells exposed to H_2_O_2_-induced oxidative stress conditions doubled compared to controls without H_2_O_2_ exposure (Figure 5). It appears that oxidative stress-induced cardiac cells increase lipid-raft signalling for the binding of EGCg. Accordingly, these rafts could function as platforms to mediate the EGCg intracellular signalling for cardioprotection against oxidative stress.

Increasing evidence indicates that multiple signal transduction events in the heart occur via caveolae and caveolins (Cavs) to localize signalling molecules and receptors in the membrane for cardioprotection [10,11,18,19]. Both Cav-1 and Cav-3, functioning as scaffolding proteins, can provide direct temporal and spatial regulation with signalling molecules activated by a wide spectrum of cardioprotective agents including the volatile anesthetic isoflurane [17,18]. Cav-1 has been shown to play a signalling role in cardiomyocytes [18]. In contrast, Cav-3, the muscle-specific isoform, mediates interactions with cytoskeletal elements and is responsible for caveolae formation in cardiac cells [42]. Several myocardial pathologies have been shown to be associated with alterations in Cav expression: Cav-1 and Cav-3 levels are elevated in pressure-overloaded and failing hearts [43,44], whereas reduced cardiac Cav-1 and Cav-3 expression has been reported in cases of myocardial infarction [45], cardiac hypertrophy [46], heart failure [47], and chronic hypoxia [48]. Cav-1 levels are also altered in renal failure [49] and pulmonary hypertension [45]. In the present study, using in vitro H_2_O_2_-induced oxidative stress in H9c2 cells, we demonstrated that H_2_O_2_ caused a 30% decrease in the levels of Cav-1 concomitant with a 20% decrease in phosphorylated Cav-1, and these reductions were counteracted by 10 or 20 μM EGCg pre-treatment for 30 min (Figure 5b). Since pre-treatment with GSK-3β inhibitor, SB 216763, also blunted the effects of H_2_O_2_ induced oxidative stress on Cav-1 inhibition (Figure 7b, c), it is very likely that EGCg could act through GSK-3β to affect Cav-1 signalling in H_2_O_2_-induced cells. The link between Cav-1 activation and GSK-3β signalling pathway could be achieved by Akt activation (Figure 7a). Thus, during oxidative stress by myocardial ischemia assault Cavs can modulate intracellular signalling for EGCg-medicated cardioprotection via Akt/GSK-3β pathway. In addition, using a rat model of myocardial ischemia involving LAD ligation, we demonstrated that GTPs treatment for 2 weeks efficiently protected infarcted myocardium of LAD-ligated rats from reduced Cav-3 protein levels (Figure 6). It appears that during oxidative injury or myocardial ischemia, Cavs can modulate intracellular signalling for GTP-medicated cardioprotection.

## Conclusions

In summary, the results reported here suggested that GTPs may mediate cardioprotection against oxidative stress and ischemic injury through caveolae trafficking via Akt/GSK-3β pathway.

## Competing interests

The authors declare that they have no competing interests.

## Authors’ contributions

HSR carried out LAD ligation and participated in the design of the study. HCS performed experiments for testing cardio protective effects of EGCg in H_2_O_2_ induced H9c2 cell injury. LCH carried out experiments in EGFP-expressed H9c2 cells. CWC performed EGFP immunoprecipitation, 2D gel analyses and molecular identification on EGFP-associated protein complexes in expressing H9c2 cells. CCH participated in animal care and GTPs-feeding. LYM conceived of the study and participated in its design and coordination and helped to draft the manuscript and final MS submission. All authors read and approved the final manuscript.

## Supplementary Material

Additional file 1: Figure S1 The mass spectra information in Figure 4c. Myosin IX [myh9, 200 kD, EDM15905], β-actin [43 kD, ABM16832)]. Click here for file
