# Supplementary figures and images for: Epigallocatechin-3-gallate-mediated cardioprotection by Akt/GSK-3β/caveolin signalling in H9c2 rat cardiomyoblasts
Source: J Biomed Sci. 2013 Nov 19;20(1):86. doi: 10.1186/1423-0127-20-86 (PMC3871020; doi:10.1186/1423-0127-20-86)

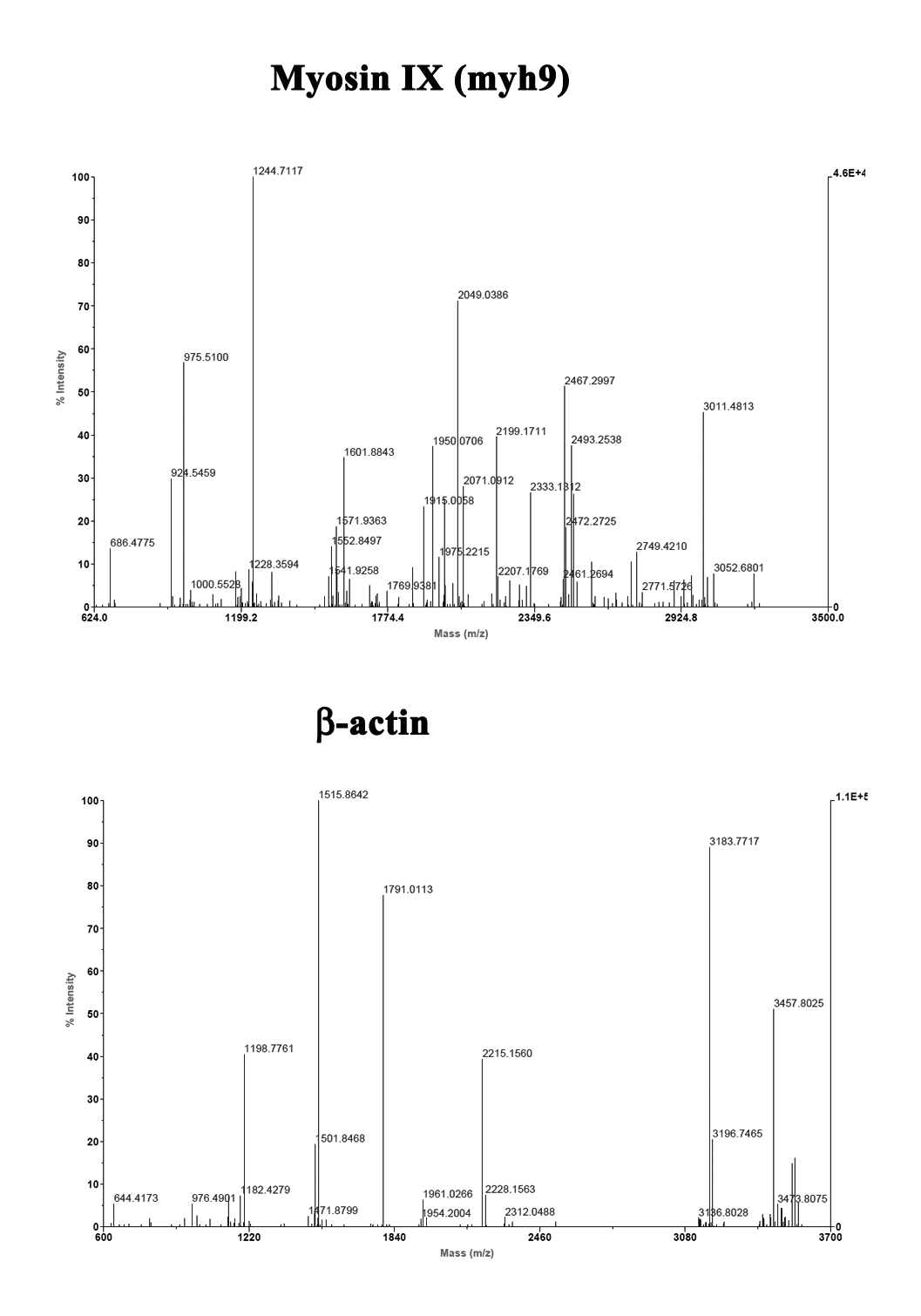

Supplement: Additional file 1: Figure S1 — The mass spectra information in Figure 4c. Myosin IX [myh9, 200 kD, EDM15905], β-actin [43 kD, ABM16832)]. [file 1423-0127-20-86-S1.tiff]
